# Multidrug-Resistant Staphylococcus aureus Isolates in a Tertiary Care Hospital, Kingdom of Bahrain

**DOI:** 10.7759/cureus.37255

**Published:** 2023-04-07

**Authors:** Abdullah AlSaleh, Mohammed Shahid, Eman Farid, Nermin Saeed, Khalid M Bindayna

**Affiliations:** 1 Microbiology, Immunology and Infectious Diseases, Arabian Gulf University, Manama, BHR; 2 Microbiology, Salmaniya Medical Complex, Manama, BHR

**Keywords:** panton-valentine leukocidin (pvl), antibiotic resistance genes, ha-mrsa, ca-mrsa, methicillin resistant staphylococcus aureus (mrsa)

## Abstract

Background: Methicillin-resistant *Staphylococcus aureus* (MRSA) is a ubiquitous pathogen associated with a wide spectrum of human infections. In recent decades, MRSA infections have been increasingly reported in individuals without established risk factors, infecting immunocompetent members of the community. This emergence is attributed to the production of various virulence factors, notably Panton-Valentine leukocidin (PVL).

Objective: The aim of this study was to better understand the prevalence, antibiotic resistance profiles, and molecular characteristics of *S. aureus* and MRSA in a tertiary care hospital in the Kingdom of Bahrain.

Materials and methods: This cross-sectional study was carried out in a tertiary hospital for a one-year period, from December 2020 to December 2021. A total of 161 consecutive *S. aureus* isolates were collected. Antibiotic susceptibility was tested using BD Phoenix™ automated identification and susceptibility testing system. Molecular analysis was conducted via conventional PCR and conventional multiplex PCR for SCC*mec* typing.

Results: In this study, 161 *S. aureus *isolates were investigated, 60% (n=97) were characterized as MRSA, of which, 12% (n=12) were healthcare-associated methicillin-resistant *Staphylococcus aureus* (HA-MRSA) while 88% (n=85) were community-associated methicillin-resistant *Staphylococcus aureus* (CA-MRSA). No statistically significant difference (P>0.05) in antibiotic resistance trends between HA-MRSA and CA-MRSA was detected. Multidrug resistance (MDR) amounted to 19% (n=30) of all *S. aureus* isolates, 14% (n=9) of methicillin-susceptible *Staphylococcus aureus* (MSSA) isolates, and 22% (n=21) of MRSA isolates. SCC*mec* typing demonstrated a high prevalence of type IV (61%, n=59), followed by type V (32%, n=31), then type II (4%, n=4), and type III (3%, n=3). The PVL prevalence was 39% (n=25) in MSSA and 62% (n=60) in MRSA, 33% (n=4) in HA-MRSA, and 66% (n=56) in CA-MRSA.

Conclusion: This study demonstrated the emergence of PVL-producing CA-MRSA in a tertiary care hospital, as well as the detection of PVL-producing MDR strains. This development prompts serious measures to be taken in order to sustain a healthy clinical environment.

## Introduction

Methicillin-resistant *Staphylococcus aureus* (MRSA) is a ubiquitous pathogen associated with a wide spectrum of human infections, ranging from mild cutaneous infections to severe systemic diseases. Historically, MRSA has been reported within healthcare facilities (HA-MRSA), infecting patients with a history of hospitalization and clinical comorbidities [1]. In recent decades, however, MRSA infections have been increasingly reported in individuals without established risk factors, leading to various recorded outbreaks in athletic teams, daycare centers, and even in some minority communities [1,2]. Indeed, reports of various community-associated (CA-MRSA) strains perpetuated globally and eventually overran the healthcare environment to become the prominent strains [3]. For example, a massive increase in CA-MRSA isolation was reported in healthcare facilities in Canada in a 10-year study [4]. Similarly, a 15-year study in Taiwan concluded that CA-MRSA strains have replaced HA-MRSA in neonatal units [5]. Also, a predominance of CA-MRSA in the skin and soft tissue infections (SSTIs) and bacteremia isolates has been reported in Australia, China, the USA, and North Africa among other regions [6-9].

CA-MRSA strains generally harbor SCCmec IV and V, the smallest of the Staphylococcal Chromosomal Cassettes (SCC) carriers of methicillin resistance gene *mecA* [10]. On the other hand, HA-MRSA strains generally harbor larger SCCmec types (I, II, and III) that have the capacity to harbor additional antibiotic resistance genes such as fusC, ermA, and tetK [10,11]. Henceforth, multidrug resistance is more common in HA-MRSA [10].

MRSA produces an armamentarium of virulence factors that may play a role in its survival and persistence [12]. Although many of these factors have unclear clinical significance, considerable attention has been centered on Panton-Valentine Leukocidin (PVL). PVL is a bi-component (LukS-PV and LukF-PV) pore-forming cytotoxin that targets the cell membrane of leucocytes, monocytes, and macrophages and is often associated with severe clinical sequelae [3]. PVL is often regarded as a marker of CA-MRSA, as it is uncommonly harbored by HA-MRSA isolates, thus, a strong epidemiological association has been postulated between PVL and the emergence of CA-MRSA in clinical settings [1]. Globally, numerous reports have been stating elevated rates of PVL-producing MRSA strains from clinical isolates including Canada (30%), Germany (40%), North Africa (20-100%), Afghanistan (71%), Palestine (30%), Myanmar (67%), the Philippines (38%), and Australia (28%) [4,6,9,13-17], which substantiates the notion that PVL-producing MRSA is an emerging healthcare phenomenon that may complicate effective clinical practices around the world. Due to the limited local data on the MRSA status, the aim of this study was to better understand the prevalence, antibiotic resistance profiles, and molecular characteristics of MRSA in a tertiary care hospital in the Kingdom of Bahrain.

## Materials and methods

Ethics statement

This study was approved by the Arabian Gulf University’s Research and Ethics Committee (REC) (approval no. E26-PI-01/20), and by the Research Technical Support Team (RTST) in the Ministry of Health, Bahrain (approval no. AURS/321/2020). Informed bilingual consent (Arabic and English) was issued for all participants, opting out of this study was a clear option for all participants. The anonymity of the data was a prime amendment in this study, and no personal information was collected.

Samples

A total of 161 consecutive, non-duplicate, *S. aureus* isolates were collected from Al-Salmaniya Medical Complex (SMC) microbiology laboratory from December 2020 to December 2021. Isolates were preserved in 10% glycerol LB broth (Sigma-Aldrich, USA) at -80°C in triplicates. The isolates were collected from various clinical samples such as pus (119), blood (20), urine (4), and tissue biopsy (10), as presented in Table 3.

Antibiotic susceptibility testing was carried out via BD Phoenix™ automated identification and susceptibility testing system using BD Phoenix™ PID panel (cat# 448008). For further confirmation, all isolates (n=161) were screened for methicillin-resistance via molecular detection of the mecA gene and cefoxitin (30µg) disc diffusion on Mueller-Hinton agar (Sigma-Aldrich, USA) in accordance with CLSI guidelines-M02 [18]. Methicillin resistance was ascertained by a zone of inhibition of ≤21mm with cefoxitin (30µg). Inducible Clindamycin Resistance (ICR) was confirmed via D-test in accordance with CLSI guidelines-M100 [19]. Clindamycin (DA, 2µg) and erythromycin (E, 15µg) discs were used.

Clinical definitions

The term ‘inpatient’ described patients that required at least an overnight stay, while the term ‘outpatient’ referred to patients visiting the hospital without a hospital stay, i.e. visiting accidents and emergency services, outpatient clinics, or same-day surgery.

The term CA-MRSA referred to MRSA strains that were isolated from outpatients, or isolated in less than 48 hours of hospitalization, or from inpatients void of MRSA common risk factors [20,21]. These risk factors include a history of recurrent MRSA infections, a history of long-term admission to a hospital or nursing home in the previous year, and a history of dialysis, permanent indwelling catheters, or other invasive medical devices [20,21].

Multidrug-resistant (MDR) strains referred to *S. aureus* isolates that were resistant to at least one agent in at least three different antimicrobial groups [22].

Molecular analysis

Conventional PCR was conducted to determine the presence of mecA, femA, nucA, and lukS/F-PV (Table 1). PCR amplification was performed with final concentrations of 0.4 µM primers, 200 µM dNTPs, 3mM MgCl2, and 500 ng of extracted bacterial DNA. PCR conditions were as follows: initial denaturation for 4 min at 94°C, followed by 30 cycles of denaturation (30s at 94°C), annealing (30s at 55°C), and extension (60s at 72°C), and a final extension for 4 min at 72°C. Gel electrophoresis was performed via Sub-Cell GT (Bio-Rad, CA, USA) on a 1.5% agarose gel and stained with ethidium bromide, afterward, the gel was visualized under ultraviolet light via UV transilluminator (BioVision, NY, USA). USA300 reference strain was used as a positive control, courtesy of the MRSA reference laboratory, Department of Microbiology, School of Medicine, Kuwait University.

**Table 1:** The primers used for molecular analysis.

Target	Primer	Sequence (5’→’3)	Produce, bp	Reference
mecA	F	AGAAGATGGTATGTGGAAGTTAG	583	[23]
	R	ATGTATGTGCGATTGTATTGC		
nucA	F	GCGATTGATGGTGATACGGTT	270	[24]
	R	AGCCAAGCCTTGACGAACTAAAGC		
femA	F	CTTACTTACTGCTGTACCTG	648	[25]
	R	ATCTCGCTTGTTGTGTGC		
LukS/F-PV	F	ATCATTAGGTAAAATGTCTGGACATGATCCA	433	[26]
	R	GCATCAASTGTATTGGATAGCAAAAGC		

SCCmec typing

**Figure 1:**
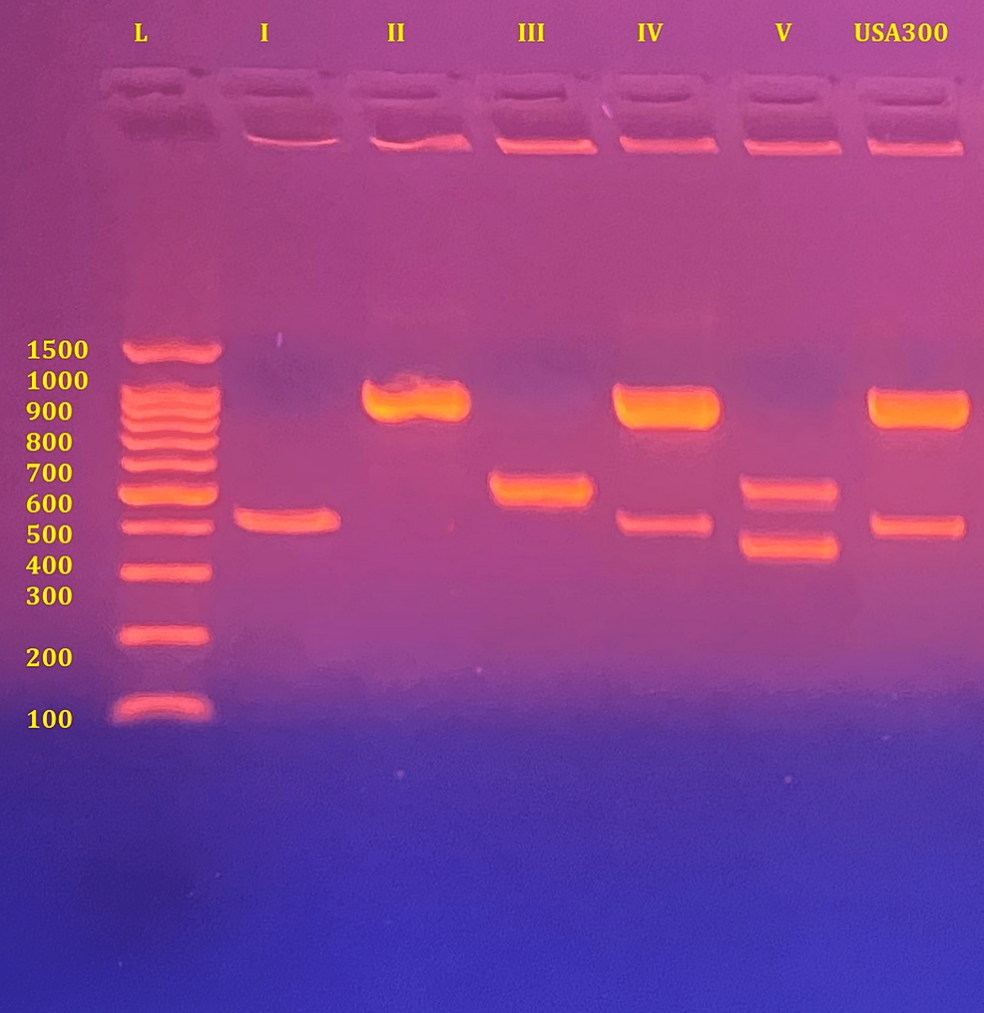
SCCmec typing for the reference strains.

SCCmec typing was carried out via multiplex PCR (Table 2) as described by Boye [23]. PCR was performed in a total volume of 25µl containing 2X PCR master mix (Promega, USA), 0.2µM of primer α3 and β each, 0.25µM of primer ccrCF and ccrCR each, and 0.1µM of primer 5RmecA and 5R431 each, in addition to 500 ng of bacterial DNA. The reaction followed these conditions: initial denaturation for 4 min at 94°C, followed by 30 cycles of denaturation (30s at 94°C), annealing (30s at 55°C), and extension (60s at 72°C), and a final extension for 4 min at 72°C. Gel electrophoresis was performed via Sub-Cell GT on a 1.5% agarose gel via ethidium bromide staining, afterward, the gel was visualized under ultraviolet light via UV transilluminator. Five MRSA reference strains were used as controls for SCCmec typing, type I: COL, type II: EMRSA-16, type III: UK-1, type IV: EMRSA-15, type V: WBG-8318 (Figure 1); courtesy of MRSA reference laboratory, Department of Microbiology, School of Medicine, Kuwait University.

**Table 2:** The primers used for SCCmec typing.

Target	Primer	Sequence (5’→’3)	Produce, bp	Reference
ccrA2-B	F	ATTGCCTTGATAATAGCCYTCT	937	[23]
	R	TAAAGGCATCAATGCACAAACACT		
ccrC	F	CGTCTATTACAAGATGTTAAGGATAAT	518	
	R	CCTTTATAGACTGGATTATTCAAAATAT		
IS1272	F	GCCACTCATAACATATGGAA	415	
	R	CATCCGAGTGAAACCCAAA		
mecA-IS431	F	TATACCAAACCCGACAACTAC	359	
	R	CGGCTACAGTGATAACATCC		

Statistical analysis

Data analysis was conducted via IBM SPSS Statistics for Windows (version 20, IBM Corp, Armonk, NY, USA). The chi-square test was used to determine significant marginal differences between HA-MRSA and CA-MRSA with regard to antibiotic sensitivity, molecular characterization, and site of collection. A p-value less than 0.05 was considered significant.

## Results

Clinical isolates

In this study, 161 *S. aureus* isolates were collected during the study period, of which 37% (n=60) were from inpatients, 59% (n=95) from outpatients, and 4% (n=6) were unspecified. Ninety-seven isolates were characterized as MRSA, 12 (12%) were identified as HA-MRSA, and 85 (88%) as CA-MRSA based on the criteria described in the Methods section. The isolates originated from various clinical samples as seen in Table 3. The participants in this study (n=161) were in the age range of 1 to 91 (mean=41), and the majority were in the age groups 40-60 (n=49, 30%) and 20-39 (n=49, 30%) followed by the age groups 1-19 (n=28,17%) and >60 (n=26, 16%).

**Table 3:** Clinical samples for Staphylococcus aureus isolates. Chi-square was used to determine significant marginal differences between HA-MRSA and CA-MRSA (p <0.05 was considered significant). MSSA: methicillin-sensitive *Staphylococcus aureus*, MRSA: methicillin-resistant *Staphylococcus aureus*, HA: healthcare-associated, CA: community-associated

Sample Type	Frequency %	MSSA (n=64) n (%)	MRSA			
			HA-MRSA (n=12) n (%)	CA-MRSA (n=85) n (%)	P-value	Total MRSA (n=97) n (%)
Pus (n=119)	74	44 (69)	9 (75)	66 (78)	>0.05	75 (77)
Blood (n=20)	12	10 (15.5)	2 (17)	8 (9)	>0.05	10 (10)
Tissue biopsy (n=10)	6	6 (9)	1 (8)	3 (3.5)	>0.05	4 (4)
Urine (n=4)	3	1 (1.5)	0 (0)	3 (3.5)	>0.05	3 (3)
Unspecified (n=8)	5	3 (5)	0 (0)	5 (6)	>0.05	5 (5)

Antibiotic susceptibility testing

All (100%) MRSA isolates were resistant to Beta lactams (cefoxitin, oxacillin, and penicillin), and were susceptible to daptomycin, doxycycline, linezolid, nitrofurantoin, quinupristin-dalfopristin, and vancomycin. CA-MRSA showed increased resistance rates over HA-MRSA to ciprofloxacin, erythromycin, norfloxacin, and trimethoprim, while HA-MRSA showed increased resistance to chloramphenicol, gentamicin, and mupirocin as seen in Table 4, however, the difference was not statistically significant (P>0.05). In regard to MSSA, higher resistance rates were recorded in both clindamycin and inducible clindamycin resistance (ICR). Macrolide-Lincosamide-streptogramin B (MLSB) resistance phenotype was detected in 6% (n=9) of all *S. aureus* isolates collected in this study, 8% (n=5) of MSSA isolates and 4% (n=4) of MRSA isolates. MDR was detected in 19% (n=30) of *S. aureus* isolates, 22% (n=21) of MRSA isolates, and 14% (n=9) of MSSA isolates (Table 5).

**Table 4:** Antibiotic resistance testing of the isolates collected in the study. BD Phoenix™ automated identification and susceptibility testing system using BD Phoenix™ PID panel was used to interpret sensitivity and resistance. Chi-square was used to determine significant marginal differences between HA-MRSA and CA-MRSA (p <0.05 was considered significant). MSSA: methicillin-sensitive *Staphylococcus aureus*, MRSA: methicillin-resistant *Staphylococcus aureus*, HA: healthcare-associated, CA: community-associated, ICR: inducible clindamycin resistance, MLSB: macrolide-lincosamide-streptogramin B resistance

Antibiotic Class	Antibiotic	MSSA (n=64) n (%)	MRSA			
			HA-MRSA (n=12) n (%)	CA-MRSA (n=85) n(%)	P-value	Total MRSA (n=97) n(%)
Cephalosporins	Cefoxitin	0 (0)	12 (100)	85 (100)	-	97 (100)
Penicillins	Penicillin G	56 (88)	12 (100)	85 (100)	>0.05	97 (100)
	Oxacillin	0 (0)	12 (100)	85 (100)	-	97 (100)
Phenicols	Chloramphenicol	2 (3)	1 (8)	7 (8)	>0.05	8 (8)
Quinolones	Ciprofloxacin	25 (39)	6 (50)	51 (60)	>0.05	56 (58)
	Moxifloxacin	1 (2)	3 (25)	13 (15)	>0.05	16 (16)
	Norfloxacin	25 (39)	4 (33)	41 (48)	>0.05	45 (46)
Lincosamides	Clindamycin	5 (8)	0 (0)	6 (7)	>0.05	6 (6)
Lipopeptides	Daptomycin	0 (0)	0 (0)	0 (0)	-	0 (0)
Tetracyclines	Doxycycline	0 (0)	0 (0)	0 (0)	-	0 (0)
Macrolides	Erythromycin	12 (19)	2 (17)	43 (51)	<0.05	45 (46)
Aminoglycosides	Gentamicin	1 (2)	4 (33)	12 (14)	>0.05	16 (16)
Oxazolidinones	Linezolid	0 (0)	0 (0)	0 (0)	-	0 (0)
Psudomonic acid	Mupirocin	0 (0)	1 (8)	2 (2)	>0.05	3 (3)
Nitroheterocyclic Agents	Nitrofurantoin	0 (0)	0 (0)	0 (0)	-	0 (0)
Streptogramins	Quinupristin-dalfopristin	0 (0)	0 (0)	0 (0)	-	0 (0)
Rifamycins	Rifampin	0 (0)	0 (0)	2 (2)	>0.05	1 (1)
Folate pathway antagonists	Trimethoprim	21 (33)	4 (33)	31 (36)	>0.05	36 (37)
	Trimethoprim-Sulfamethoxazole	21 (33)	2 (17)	27 (32)	>0.05	29 (30)
Glycopeptides	Teicoplanin	0 (0)	0 (0)	0 (0)	-	0 (0)
	Vancomycin	0 (0)	0 (0)	0 (0)	-	0 (0)
Resistance Phenotypes	ICR	15 (23)	0 (0)	17 (20)	-	17 (18)
	MLSB	5 (8)	0 (0)	4 (5)	-	4 (4)

**Table 5:** The resistance profile, SCCmec type, and PVL acquisition of MDR strains collected in this study. BD Phoenix™ automated identification and susceptibility testing system using BD Phoenix™ PID panel was used to interpret sensitivity and resistance. Multiplex PCR was used for SCCmec typing. Conventional PCR was used to determine PVL acquisition. MDR: multi-drug resistance, PVL: Panton-Valentine Leukocidin, CHL: chloramphenicol, CIP: ciprofloxacin, CLI: clindamycin, ERY: erythromycin, GEN: gentamicin, ICR: inducible clindamycin resistance, MUP: mupirocin, MXF: moxifloxacin, NOR: norfloxacin, SXT: trimethoprim/sulfamethoxazole, TMP: trimethoprim

Combo	ID	MDR strains (n=30) n (%)	Resistance profile	SCCmec type	PVL
1	MRSA	2 (7%)	CIP, MXF, NOR, ERY, GEN, TMP, SXT	V	+ve
2	MRSA	1 (3%)	CIP, MXF, NOR, ICR, ERY, TMP	V	-ve
3	MRSA	1 (3%)	CIP, MXF, NOR, ERY, MUP	II	+ve
4	MRSA	2 (7%)	CIP, MXF, NOR, CLI, ERY, TMP	IV	+ve
5	MRSA	1 (3%)	CHL, CLI, ERY	IV	-ve
6	MRSA	10 (33%)	CIP, MXF, NOR, ERY, TMP, SXT	IV, V	+ve
7	MRSA	3 (10%)	CIP, MXF, NOR, GEN, TMP, SXT	IV, V	+ve
8	MRSA	1 (3%)	CHL, CIP, MXF, NOR, TMP	V	-ve
9	MSSA	2 (7%)	CHL, CIP, MXF, NOR, CLI, ERY, TMP, SXT	-	-ve
10	MSSA	2 (7%)	CIP, MXF, NOR, CLI, ERY	-	-ve
11	MSSA	2 (7%)	CIP, MXF, NOR, ICR, ERY, TMP, SXT	-	-ve
12	MSSA	1 (3%)	CHL, CIP, MXF, NOR, CLI, ERY, TMP, SXT	-	+ve
13	MSSA	2 (7%)	CIP, MXF, NOR, ICR, ERY, TMP, SXT	-	+ve

Molecular characterization

As shown in Table 6, all MRSA isolates harbored mecA and nucA, whereas femA was harbored by 58% and 78% of MSSA and MRSA isolates respectively. The SCCmec typing results showed that predominantly CA-MRSA expressed SCCmec types IV (68%, n=58) and V (32%, n=27), whereas HA-MRSA predominantly harbored SCCmec types II (33%, n=4), III (25%, n=3), and V (33%, n=4). PVL detection in MSSA was 39% (n=25) and 62% (n=60) in MRSA, furthermore, the majority of CA-MRSA were PVL-positive (66%, n=56) while detection in HA-MRSA was less at 33% (n=4) belonging to SCCmec types II and III.

**Table 6:** Molecular characteristics of the study isolates. Multiplex PCR was used in SCCmec typing. Chi-square was used to determine significant marginal differences between HA-MRSA and CA-MRSA (p <0.05 was considered significant). MSSA: methicillin-sensitive *Staphylococcus aureus*, MRSA: methicillin-resistant *Staphylococcus aureus*, HA: healthcare-associated, CA: community-associated

Target	MSSA (n=64) n (%)	MRSA			
		HA-MRSA (n=12) n (%)	CA-MRSA (n=85) n (%)	P-value	Total MRSA (n=97) n (%)
mecA	0 (0)	12 (100)	85 (100)	-	97 (100)
nucA	64 (100)	12 (100)	85 (100)	-	97 (100)
femA	37 (58)	7 (58)	69 (81)	>0.05	76 (78)
Luk-PV	25 (39)	4 (33)	56 (66)	<0.05	60 (62)
SCCmec Type:					
I	-	0 (0)	0 (0)	-	0 (0)
II	-	4 (33)	0 (0)	<0.05	4 (4)
III	-	3 (25)	0 (0)	<0.05	3 (3)
IV	-	1 (8)	58 (68)	<0.05	59 (61)
V	-	4 (33)	27 (32)	>0.05	31 (32)

## Discussion

MRSA is a versatile bacterial pathogen that may infect immunocompromised patients in healthcare settings (HA-MRSA) as well as immunocompetent individuals in the community (CA-MRSA) [3]. Because of unknown reasons, CA-MRSA strains have emerged and propagated substantially into hospitals and healthcare centers [3]. This emergence has the potential to further complicate staphylococcal infections as CA-MRSA strains generally harbor virulence factors not expressed in the HA-MRSA strains [3].

Molecular characterization

SCCmec typing illustrated a dominance for type IV at 61% (n=59) followed by type V at 32% (n=31), while types II and III comprised 7% (n=4 and 3 respectively). This clear prominence of type IV has been demonstrated regionally, as the most dominant SCCmec type was type IV in most reports from Gulf Council Cooperation (GCC) countries (19-90%) over the past 10 years, as well as in Iran over 58-71% [24-26]. Locally, in a 2008 study conducted on inpatients hospitalized in SMC, the vast majority of MRSA isolates harbored SCCmec type III (86.7%), and the rest were type IV (13.3%) [27]. Conversely, MRSA isolates from inpatients in this study majorly harbored types IV and V at 63% (n=22) and 29%(n=10) respectively, while type III only comprised 3% (n=1), which may be attributed to the recent emergence of CA-MRSA into healthcare settings.

Furthermore, we reported a high prevalence of CA-MRSA at 88% (n=85) amongst MRSA isolates. These community-associated strains harbor SCCmec types IV and V at rates of 68% (n=58) and 32% (n=27) respectively as seen in Table 6. This emergence has been reported regionally to higher levels, as in Kuwait (91%), Oman (91%), Qatar (95%), and United Arab Emirates (UAE) (98%), in fact, most SCCmec typing reports in the GCC report a dominance in type IV CA-MRSA ranging from 19 to 90% [24,28-31]. The reason for this global phenomenon is not well-understood but can be attributed to improved genomic fitness and the ability to produce a large spectrum of virulence factors [32].

PVL is a complex staphylococcal exotoxin associated with high morbidity and mortality owing to its varying leukocytoclastic and dermonecrotic properties [1]. In this study, PVL was detected in MSSA and MRSA isolates at a prevalence of 39% (n=25) and 62% (n=60) respectively as seen in Table 6. PVL-producing MRSA strains were isolated mostly from pus (82%, n=49), blood (7%, n=4), and tissue biopsy (4%, n=2), similarly, PVL-producing MSSA strains were isolated mostly from pus (80%, n=20), blood (8%, n=2), and skin biopsy (8%, n=2). Moreover, outpatient isolates amounted to 69% (n=42) of PVL-producing MRSA and 85% (n=21) of PVL-producing MSSA. PVL-producing MRSA prevalence reported in this study is higher than average regional rates at 35-45%, nevertheless, few prevalence reports from nearby countries have detected comparable rates as in Qatar at 66%, Saudi Arabia at 59%, and Iran at 53% [24,25,30,33]. Similarly, PVL-producing MSSA rates reported in this study were higher than regional reports for example in a tertiary hospital in Kuwait PVL was detected in 12% of MSSA isolates, furtherly asserting the regional emergence of PVL in healthcare facilities [34].

In this study, the recorded prevalence of PVL in CA-MRSA was 66% (n=56) as seen in Table 6. This high rate is not surprising because, historically, PVL has been associated with the pathophysiology of community-associated strains, in fact, it was often considered a marker for CA-MRSA [1]. Interestingly, PVL was detected in 33% (n=4) of HA-MRSA strains that also harbored SCCmec II and III as seen in Table 6. This mobility of virulence genes across MRSA types may increase the morbidity of nosocomial infections caused by HA-MRSA when accompanying antibiotic drug resistance with aggressive pathology, consequently, further complicating effective chemotherapy measures [10,24]. Alarmingly, PVL-producing HA-MRSA strains have also been reported in regional reports for example in Qatar (7%), Kuwait (28%), and Saudi Arabia (63%), further cementing the dangers of MRSA infections in the region [30,33,35].

Antibiotic susceptibility testing

In this study, 21 antibiotics were used to test the resistance profiles of MRSA isolates in Bahrain. Resistance to non-beta-lactam antibiotics has been detected, notably against ciprofloxacin (58%), erythromycin (46%), norfloxacin (46%), trimethoprim (37%), and trimethoprim-sulfamethoxazole (30%) as seen in Table 4. Despite resistance rates being within the regional ranges, they show a substantial decline from a previous report from 2008 that reported overall higher antibiotic resistance rates in MRSA isolated from SMC [24,27]. This favorable decline may be attributed to the implementation of the “Antibiotic Resistance and Antibiotic Stewardship Program” in SMC in 2010 which mainly concentrated on educating healthcare providers on the dangers of antibiotic resistance and the liberal prescription of antimicrobials without microbiological evidence, however, more investigations are needed to ascertain the efficacy of this program.

It is worth noting that ICR was found in 20% (n=32) of all isolates, specifically, 18% (n=17) of MRSA isolates all of which were community-associated. Moreover, 4% (n=4) of MRSA isolates expressed constitutive MLSB phenotype, all of them also were CA-MRSA belonging to SCCmec type IV, also, 8% (n=5) of MSSA isolates expressed constitutive MLSB phenotype. Similar ICR rates were reported regionally, for example, ICR prevalence was reported to be 20% in both Qatar and Oman and 17% in Kuwait, while lower rates were reported in Iran at 13% [26,29,30,36]. However, higher rates of constitutive MLSB were reported regionally as in Kuwait at a rate of 16% and Iran at a high rate of 53% [26,36].

Contrary to expectations from the literature, no significant difference (P>0.05) was observed between HA-MRSA and CA-MRSA resistance profiles, except when comparing erythromycin resistance (P<0.05) as seen in Table 4. It is well established that HA-MRSA strains exhibit antimicrobial resistance more so than CA-MRSA, however, with the prominence and persistence of CA-MRSA in healthcare facilities and with the permissive gene exchange among MRSA strains, this differentiation may not be as distinct as previously believed [1,10]. All in all, more studies are required to establish this new trend, particularly with larger sample sizes.

MDR *S. aureus* strains are defined to be resistant to at least three different groups of antibiotics [22]. In our study, 19% (n=30) of all *S. aureus* isolates, 14% (n=9) of MSSA isolates, and 22% (n=21) of MRSA isolates were categorized as MDR, mostly expressing resistance against antimicrobial groups like quinolones, macrolides, and folate pathway antagonists, as seen in Table 5. Comparatively, MDR rates from the 2008 local article reported a higher prevalence at 89% of MRSA isolates and most of them harbored SCCmec type III [27]; whereas most of our MDR strains expressed SCCmec V and IV. While the resistance patterns reported in this study differ from reported patterns in 2008, some were similar to profiles reported in other studies from Kuwait and Saudi Arabia belonging to the MRSA clones ST22-IV (Barnim/UK-EMRSA-15) and ST772-V (Bengal Bay Clone) [35,37-39]; in particular, the combinations 1, 2, 5, 6, and 7 from Table 5. It is worth noting that 70% (n=21) of MDR strains described in this study harbored the toxin PVL; so, despite the reduction in MDR rates, this emergence of PVL in MDR strains may potentially further complicate clinical treatment and maintenance.

## Conclusions

This study reported a prevalence of 88% for CA-MRSA in SMC, Bahrain, as well as the prevalence of PVL-producing MRSA and MSSA strain (62% and 39%, respectively). A clear upward trend has been illustrated in the dissemination of CA-MRSA and PVL-producing strains. With the combined efforts of the infection control department and the antibiotic stewardship committee, stricter measures may be implemented to prevent the colonization of MDR strains in healthcare facilities and to mitigate the propagation of PVL-producing CA-MRSA strains.
